# Investigating PEGDA and GelMA Microgel Models for Sustained 3D Heterotypic Dermal Papilla and Keratinocyte Co-Cultures

**DOI:** 10.3390/ijms22042143

**Published:** 2021-02-21

**Authors:** Justin J.Y. Tan, Duc-Viet Nguyen, John E. Common, Chunyong Wu, Paul C.L. Ho, Lifeng Kang

**Affiliations:** 1 Department of Pharmacy, National University of Singapore, Lower Kent Ridge Road, 18 Science Drive 4, Singapore 117543, Singapore; justin_tan@sutd.edu.sg (J.J.Y.T.); paul.ho@nus.edu.sg (P.C.L.H.); 2 Nusmetics Pte. Ltd., i4 Building, 3 Research Link, Singapore 117602, Singapore; ducvietnguyen@nusmetics.com; 3 Skin Research Institute of Singapore, Immunos, 8A Biomedical Grove, Singapore 138648, Singapore; john.common@sris.a-star.edu.sg; 4 Department of Pharmaceutical Analysis, China Pharmaceutical University, 24 Tongjiaxiang, Nanjing 210009, China; cywu@cpu.edu.cn; 5 School of Pharmacy, University of Sydney, Pharmacy and Bank Building A15, Sydney, NSW 2006, Australia

**Keywords:** epithelial-mesenchymal interaction (EMI), poly (ethylene glycol) diacrylate (PEGDA), gelatin methacrylate (GelMA), hair follicle morphogenesis, keratinocytes, dermal papilla

## Abstract

Hair follicle morphogenesis is heavily dependent on reciprocal, sequential, and epithelial-mesenchymal interaction (EMI) between epidermal stem cells and the specialized cells of the underlying mesenchyme, which aggregate to form the dermal condensate (DC) and will later become the dermal papilla (DP). Similar models were developed with a co-culture of keratinocytes and DP cells. Previous studies have demonstrated that co-culture with keratinocytes maintains the in vivo characteristics of the DP. However, it is often challenging to develop three-dimensional (3D) DP and keratinocyte co-culture models for long term in vitro studies, due to the poor intercellular adherence between keratinocytes. Keratinocytes exhibit exfoliative behavior, and the integrity of the DP and keratinocyte co-cultured spheroids cannot be maintained over prolonged culture. Short durations of culture are unable to sufficiently allow the differentiation and re-programming of the keratinocytes into hair follicular fate by the DP. In this study, we explored a microgel array approach fabricated with two different hydrogel systems. Using poly (ethylene glycol) diacrylate (PEGDA) and gelatin methacrylate (GelMA), we compare their effects on maintaining the integrity of the cultures and their expression of important genes responsible for hair follicle morphogenesis, namely *Wnt10A*, *Wnt10B*, and *Shh*, over prolonged duration. We discovered that low attachment surfaces such as PEGDA result in the exfoliation of keratinocytes and were not suitable for long-term culture. GelMA, on the hand, was able to sustain the integrity of co-cultures and showed higher expression of the morphogens overtime.

## 1. Introduction

Hair follicle morphogenesis is heavily dependent on reciprocal, sequential, epithelial-mesenchymal interaction (EMI) between epidermal stem cells and the specialized cells of the underlying mesenchyme, which aggregate to form the dermal condensate (DC) and will later become the dermal papilla (DP) [1]. Since the self-aggregation of these condensed mesenchymal cells underlying the epithelial hair germ cells (placode) in the embryonic skin is important to give rise to the DP, self-aggregation of the DP becomes an essential feature for hair follicle morphogenesis in vivo [2,3]. In a recent study published by Higgins et al., changes to the transcriptome profile were observed between freshly isolated native human DPs and DP cells cultured in monolayers for several passages, and many of these transcripts were involved in hair follicle development, implying that human DP cells lose their inductivity almost immediately upon seeding into a culture dish [4]. These expressions were partially restored when they attempted to culture the human DP cells in hanging drop cultures to create a microenvironment favoring self-aggregation to form DP spheroids. In most instances, 3D DP monocultures are cultured with the intention of transplanting the DP aggregates into animal models for in vivo studies to assess the inductivity of the DP spheroids [4,5,6]. Similar models were developed with a co-culture of keratinocytes and DP cells. Previous studies have demonstrated that co-culture with keratinocytes maintains the in vivo characteristics of the DP [7,8,9]. Qiao et al. have shown that *in vitro*-aggregated mouse follicular cells (DP cells and keratinocytes) developed partially formed hair structures (’proto-hairs’) while in culture within methylcellulose coated wells [10]. Upon implantation into nude mice via incision, the proto-hairs became mature hairs and persisted for at least 6 months. Havlickova et al. reported the use of a mixture of rat collagen I and Matrigel Basement Membrane Matrix for the preparation of a human folliculoid microsphere (HFM) consisting of outer root sheath keratinocyte (ORSK) and DP cells [11]. This model allowed for the close interaction between the ORSK and DP cells, exhibited hair follicle type keratinization (Cyto-Keratin 6) and displayed expression of versican, a chondroitin sulfate proteoglycan essential in hair follicle formation.

In addition to keratinocyte influence, cellular compartmentalization is also key to optimizing EMI crucial for hair follicle morphogenesis. Previous in vivo hair follicle reconstitution assays have demonstrated that co-grafted dissociated epithelial and dermal cells re-position themselves spontaneously to imitate their anatomical relationship in vivo, suggesting the importance of the positional relationship between epithelial and mesenchymal cells in hair follicles towards follicular morphogenesis and EMI [12]. Thus, artificially assembling cell composites mimicking the hair follicle bulb structure instead of using dissociated cells has been proposed to enhance EMIs. Pan et al. fabricated micro-structured poly (ethylene glycol) diacrylate (PEGDA) hydrogels resembling the physiological architecture of hair follicles, in which the dermal cells were encapsulated within the gel compartment and separated from the epidermal cell populations [13]. Similarly, Lim et al. developed a method to assemble DP and normal human epidermal keratinocytes (NHEK) in close proximity within 3D fibrous hydrogel scaffolds using two oppositely charged polyelectrolyte solutions [14]. This method encourages cells cultured within one domain to establish interactions amongst themselves, and with the second cell type in the neighboring domain, thereby improving EMI.

However, it is often challenging to develop 3D DP and keratinocyte co-culture models for long term in vitro studies, due to the poor intercellular adherence between keratinocytes. Keratinocytes exhibit exfoliative behavior, and the integrity of the DP and keratinocyte co-cultured spheroids cannot be maintained over prolonged culture [15]. Yen et al. have also reported that keratinocytes were non-aggregative in their study, which inhibited the formation of epithelial aggregates, thereby decreasing the cellular interactions with the DP cells [16]. Short durations of culture are unable to sufficiently allow differentiation and re-programming of the keratinocytes into hair follicular fate by the DP.Gelatin, the degradation product of collagen, is one of the many natural biopolymers which mimic natural extracellular microenvironments and has been shown to enhance cellular responses and tissue regeneration [17,18]. Gelatin contains many arginine-glycine-aspartic acid (RGD) sequences that are important for cell attachment and act as target sequences of matrix metalloproteinase (MMP) useful for cell re-modelling [19,20]. Additionally, introducing methacryloyl substituent groups to gelatin allows them to partake in photo-crosslinking in the presence of a photoinitiator and light exposure, thereby photopolymerizing the methacryloyl substituents [21]. Gelatin methacrylate (GelMA) hydrogels have been extensively studied and applied in fields ranging from tissue engineering to drug and gene delivery [21]. However, its influence on in vitro hair follicle engineering is not well understood.

In this study, we explored a microgel array approach, usingtwo different hydrogel systems, namely PEGDA and GelMA. We compared the effects of the microstructured hydrogels on maintaining the integrity and the expression of important genes responsible for hair follicle morphogenesis, namely *Wnt10A*, *Wnt10B*, and *Shh*, of the DP and human calcium- and temperature-dependent (HaCaT) keratinocyte heterotypic spheroids over prolonged duration of culture. 

## 2. Results

### 2.1. Sequential Seeding of Cells into PEGDA/GelMA Hydrogel Microwells Yields Heterotypic Spheroids of Different Configurations

**Figure 1:**
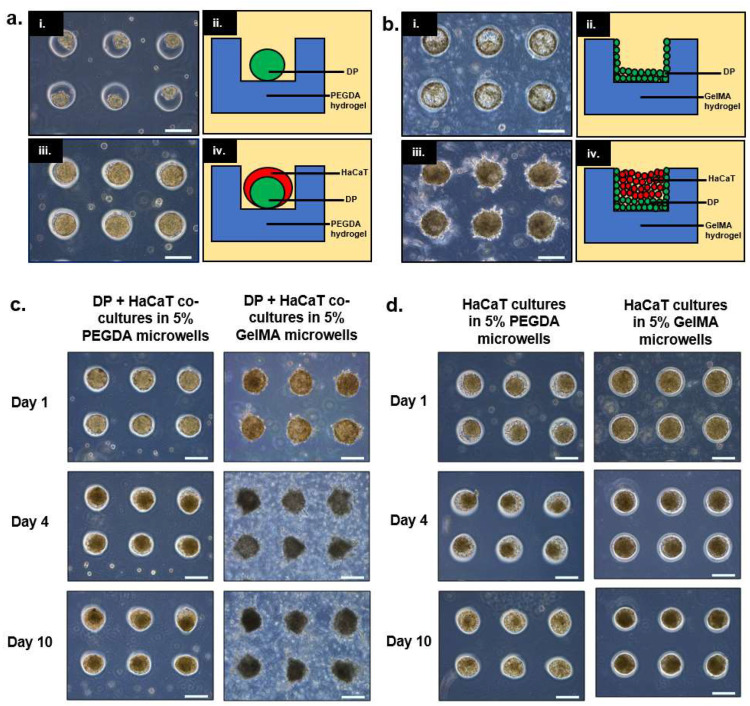
Sequential seeding of dermal papilla (DP) and HaCaT cells into poly (ethylene glycol) diacrylate (PEGDA) or gelatin methacrylate (GelMA) microwells yielded heterotypic cultures of different configurations. Exfoliation of HaCaT keratinocytes was observed in PEGDA microwells in both co-cultures and mono-cultures (**a**(i)–(iv)). Light microscopic images showing DP and DP-HaCaT keratinocyte co-culture behavior and their corresponding schematic representation. DP aggregates were formed 24 h post-seeding into PEGDA microwells. Subsequently, HaCaT keratinocytes were seeded on top of the DP aggregates to yield co-cultured aggregates 24 h later (**b**(i)–(iv)). Light microscopic images showing DP and DP-HaCaT keratinocyte co-culture behavior and their corresponding schematic representation. DP attached and spread on the base and the walls of the GelMA microwells 24 h post-seeding. Subsequently, HaCaT keratinocytes were seeded on top of the DP to yield co-cultured aggregates 24 h later (**c**). Light microscopic images of DP and HaCaT spheroids in 5% PEGDA or 5% GelMA microgels at day 1, 4, and 10, respectively (**d**). Light microscopic images of HaCaT cultures in 5% PEGDA or 5% GelMA microgels at day 1, 4, and 10, respectively. All scale bars represent 200 μm.

To produce DP-HaCaT keratinocyte composite spheroids, DP and HaCaT cells were seeded sequentially, DP first, followed by HaCaT keratinocytes 24 h post-seeding of DP cells into PEGDA or GelMA microwells to achieve compartmentalization. In the case of PEGDA microwells, DP cells displayed a non-adherent character and repelled the gel surface, forming compact aggregates 24 h post-seeding. However, we observed that the DP cells were adherent towards GelMA surfaces, hence, attaching themselves to the base and the walls of the GelMA microwells. The HaCaT keratinocytes were subsequently seeded on top of the DP cells in both the PEGDA and GelMA microwells to form composite spheroids 24 h post-seeding of HaCaT keratinocytes. Figure 1a,b illustrate the stages of sequential seeding and their corresponding cross-section schematic representation for both PEGDA and GelMA microwells, respectively.

Our co-culture model in PEGDA microgels supports relatively good aggregation of the HaCaT keratinocytes around the DP aggregate core. Unfortunately, the integrity of the co-cultured aggregate does not maintain over long periods of culture, as exfoliation of keratinocytes would have occurred after a couple of days.

### 2.2. Exfoliation of HaCaT Keratinocytes Observed in PEGDA Hydrogel Microwells in Both Co-Cultures and Mono-Cultures

The DP-HaCaT spheroids in PEGDA and GelMA microwells were kept in culture for up to 10 days, and they were observed on day 1, day 4, and day 10 under the light microscope. Figure 1c illustrates the co-cultures in PEGDA microwells undergoing significant reduction in their sizes as compared to the aggregates inside GelMA microwells over a duration of 10 days. Figure 1d illustrates the behavior of the HaCaT keratinocytes seeded within GelMA and PEGDA microwells, respectively. In the case of PEGDA microwells, it was shown that HaCaT keratinocytes exfoliated and left the microwells, owning to their poor intercellular adherence, and a substantial reduction in the size of the aggregates was observed over a period of 10 days.

### 2.3. PEGDA Hydrogels Promotes Aggregation of HaCaT Keratinocytes but Not GelMA

**Figure 2:**
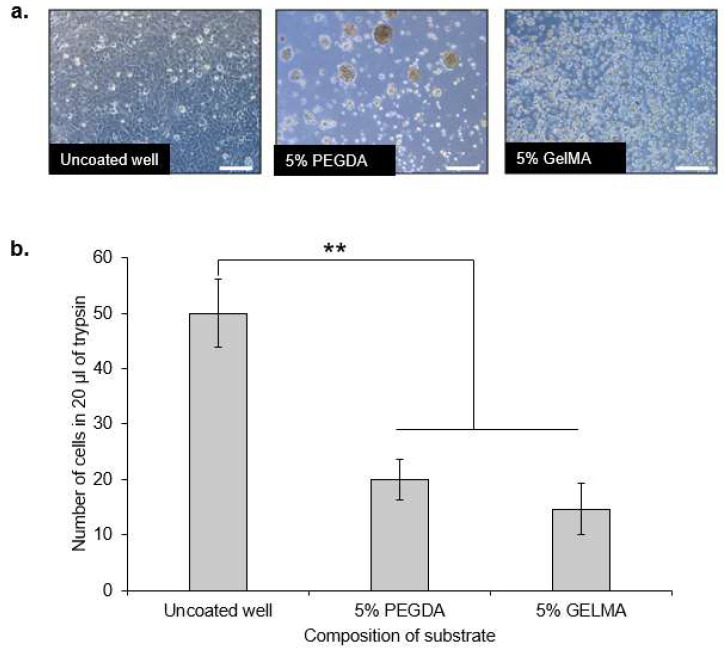
Behavior of HaCaT keratinocytes on PEGDA and GelMA hydrogels (**a**). PEGDA encourages aggregation of HaCaT keratinocytes while GelMA does not encourage aggregation of HaCaT keratinocytes, while the uncoated surface of the petri dish is used as a reference for full attachment and spreading of HaCaT keratinocytes (**b**). HaCaT keratinocytes do not differ in surface adhesiveness towards PEGDA or GelMA substrates and show preferential attachment to the uncoated wells (** *p* <0.01, as compared to the uncoated well). All scale bars represent 200 μm.

The behavior of HaCaT keratinocytes on both PEGDA and GelMA was then studied. Figure 2a illustrates the differential behavior of HaCaT keratinocytes when they were cultured above 5% PEGDA, 5% GelMA hydrogels, or uncoated wells in a petri dish. It was observed that HaCaT keratinocytes exhibited greater aggregation behavior when cultured atop PEGDA hydrogel. On pure GelMA, however, aggregation of HaCaT keratinocytes was not clearly observed and the keratinocytes were greatly dispersed over the GelMA surface. The image of the uncoated well was used as a reference for the full attachment and spreading of the HaCaT keratinocytes. It was observed that GelMA does not promote aggregation of HaCaT keratinocytes as much as PEGDA hydrogel.

### 2.4. HaCaT Keratinocytes Do Not Differ in Surface Adhesiveness towards PEGDA or GelMA

The extent of attachment of HaCaT keratinocytes towards PEGDA or GelMA surfaces was determined by washing off the HaCaT keratinocytes which were not attached to the surfaces 24 h post-seeding with phosphate buffer saline (PBS). Subsequently, the cells left on the surfaces were trypsinized and counted. More cells counted indicates a greater cell attachment towards the surface of interest. The uncoated well was used as a reference, showing the greatest number of HaCaT keratinocytes attached to the surface as compared to PEGDA and GelMA. There was no significant difference in the number of cells counted for those cultured on 5% PEGDA and 5% GelMA, while the number of cells counted for both were significantly lower (*p* <0.01) than that of the uncoated well (Figure 2b).

### 2.5. 3D Distributions of GFP-Expressing DP Cells and RFP-Expressing HaCaT Cells in PEGDA and GelMA Hydrogel Microwells

**Figure 3:**
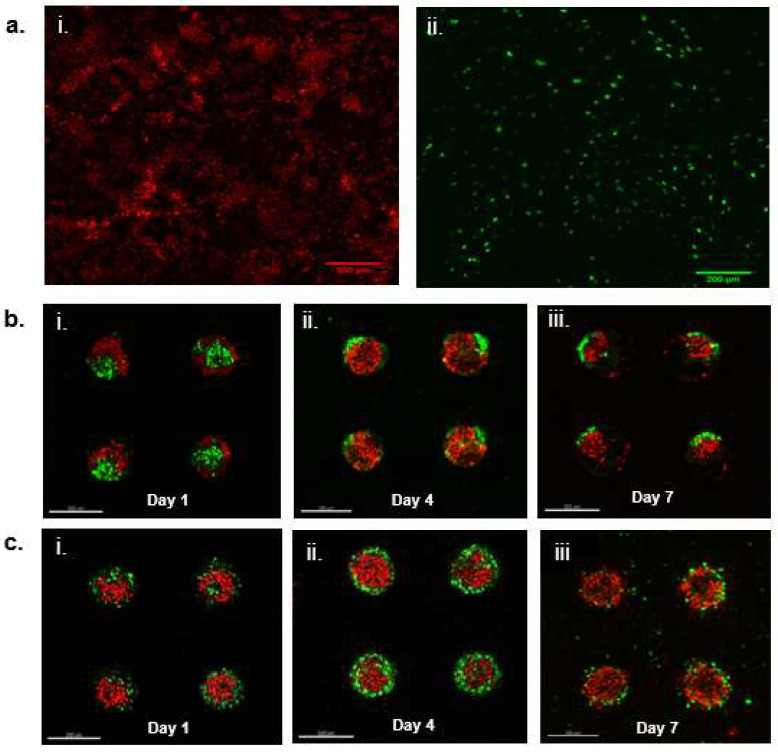
Distributions of green fluorescent protein (GFP)-expressing DP cells and red fluorescent protein (RFP)-expressing HaCaT keratinocytes in 3D cultures (**a**). Images showing fluorescence signals retained in (i) RFP-expressing HaCaT and (ii) GFP-expressing DP (**b**). Confocal images showing (i) 3D PEGDA microwells containing GFP-expressing DP surrounded by RFP-expressing HaCaT after day 1 of co-incubation. (ii) Shrinking of GFP-expressing DP aggregate on day 4 of co-incubation. (iii) Exfoliation of RFP-expressing HaCaT from co-cultured aggregates on day 7 of incubation of co-incubation (**c**). Confocal images showing (i) 3D GelMA microwells containing GFP-expressing DP surrounding RFP-expressing HaCaT after day 1 of co-incubation. (ii) GFP-expressing DP cells surrounding RFP-expressing HaCaT on day 4 of co-incubation. (iii) RFP-expressing HaCaT keratinocytes retained inside the microwells on day 7 of co-incubation. All scale bars represent 200 μm.

The distributions of HaCaT keratinocytes and DP cells within PEGDA and GelMA microwells were visualized using red fluorescent protein (RFP)-expressing HaCaT keratinocytes and green fluorescent protein (GFP)-expressing DP cells. Figure 3a shows pure cultures of RFP-expressing HaCaT keratinocytes and GFP-expressing DP cells after these cells were sorted from non-expressing cells with flow cytometry. Figure 3b shows the changes to the DP-HaCaT heterotypic spheroids while cultured in PEGDA microwells over a period of 7 days. Generally, it was observed that the DP cells have reduced in numbers or were compacted to the side of the microwells on day 4 as compared to day 1. Exfoliation of HaCaT keratinocytes from the spheroids was observed on day 7, accounting for the reduction in the sizes of the spheroids. On the other hand, Figure 3c shows the that the DP cells were distributed mainly around the walls of the GelMA microwells, with the HaCaT keratinocytes distributed at the center of the microwells. There were no observable changes to the size and the integrity of the spheroids within the GelMA microwells, indicating that the extent of exfoliation of HaCaT keratinocytes were lesser than that observed in PEGDA microwells.

### 2.6. Expressions of Wnt10A, Wnt10B and Shh Vary within DP and HaCaT Co-Cultures within PEGDA and GelMA Microwells over Time

**Figure 4:**
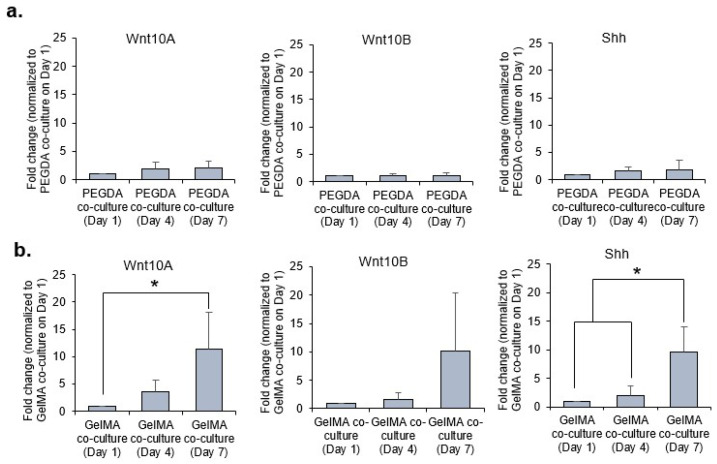

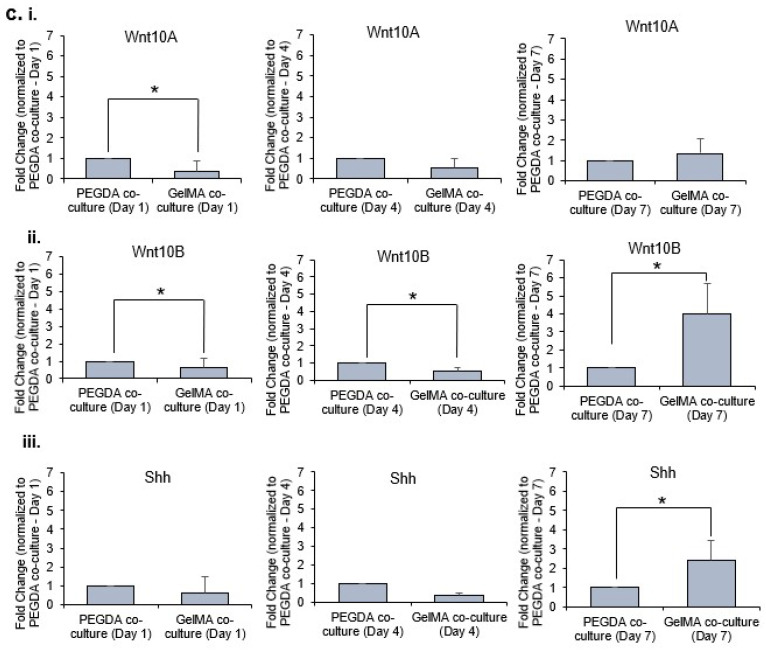
Expression of EMI-related genes (morphogens) of 3D co-cultures within PEGDA and GelMA microwells on day 1, day 4, and day 7 of incubation (**a**). There were no significant changes in the expression of morphogens on day 7 as compared to day 1 for the co-culture within PEGDA microgels (**b**). Expression of morphogens were higher on day 7 as compared to day 1 of co-culture within GelMA microgels (* *p* < 0.05, as compared to day 1 co-cultures in GelMA microwells) (**c**). Comparing the expression of morphogens between co-cultures in PEGDA and GELMA microwells on day 1, day 4, and day 7 for (i) *Wnt10A*, (ii) *Wnt10B*, and (iii) *Shh* (* *p* < 0.05 as compared to co-cultures in PEGDA microwells).

Hair follicle development is controlled by a series of reciprocal EMI. Important molecular signals mediate epithelial-mesenchymal crosstalk that drives differentiation, including members of the *Wnt* and *Shh* families of morphogens. *Wnt10a* and *Wnt10b* are the earliest *Wnt* genes to be expressed during hair morphogenesis and are essential for initiating hair development [22]. Several genes reported to induce hair follicle morphogenesis or regulate hair follicle development were investigated, including *Wnt10a*, *Wnt10b* and *Shh*. Real-time polymerase chain reaction (PCR) revealed that transcripts of genes *Wnt10a*, *Wnt10b*, and *Shh* were elevated in 3D co-cultures within GelMA microwells over time but not within PEGDA microwells, on day 7 as compared to day 1, as shown in Figure 4a,b. The increase in the gene expression was greater in 3D co-cultures within GelMA as compared to PEGDA microwells over time. Figure 4c(i)–(iii) compares the gene expression of the morphogens at respective time points between co-cultures in PEGDA and GelMA microwells. It was observed that the co-cultures within PEGDA microwells expressed greater expression of the morphogens as compared to those in GelMA microwells on day 1. Overtime, the expression of the morphogens was lower for co-cultures in PEGDA as compared to GelMA microwells on day 7.

## 3. Discussion

In this paper, we demonstrated two possible combinations of culturing epithelial (i.e., HaCaT keratinocytes) and dermal cells (i.e., DP) in PEGDA and GelMA microwells. For PEGDA microwells, it was observed that both cell types exhibited relatively low attachment to the gel surfaces, resulting in the initial aggregation of DP cells followed by the HaCaT keratinocytes to yield a core-shell configuration. On the contrary, it was observed that DP cells were adherent towards GelMA surfaces, hence forming a layer on the walls and the base of the microwells 24 h post-seeding. A similar configuration was attempted by Pan et al., whereby the mesenchymal cells (i.e., human dermal fibroblasts (HDF)) were compartmentalized within the PEGDA hydrogel scaffold while the epithelial cells (i.e., HaCaT) were seeded into the microwells made from HDF-encapsulated PEGDA hydrogels [13]. However, the difference between these two models lies in the availability of the mesenchymal component to interact with the HaCaT keratinocytes. The mesenchymal component in Pan’s model (i.e., HDF) would have been more restricted as cells were encapsulated within the gel as compared to our current model where the mesenchymal component (i.e., DP cells) was directly coating the base and the walls of the GelMA microwells, due to its adhesive nature towards the surfaces. When cultured over prolonged duration (i.e., up to 10 days), we observed that the sizes of the heterotypic spheroids within PEGDA microwells were reduced drastically, while the sizes of the aggregates within GelMA microwells remained relatively constant.

We hypothesized that the reduction in the sizes of the co-cultures within PEGDA microwells was mainly attributed to the exfoliation of HaCaT keratinocytes, owning to their overall poor intercellular adhesion, as reported in previous literature [15,16]. Therefore, we decided to investigate the behavior of HaCaT keratinocyte inside both PEGDA and GelMA microwells. As expected, the HaCaT aggregates reduced in size as the HaCaT keratinocytes exfoliated and left the microwells after 10 days of culture within the PEGDA microwells. Little changes were observed inside the GelMA microwells containing only HaCaT keratinocytes. We hypothesized that the HaCaT keratinocytes remained within the microwells, due to better attachment. On the contrary, HaCaT keratinocytes did not differ significantly in terms of substrate adhesiveness, as the number of cells harvested from the two different gel surfaces 24 h post-incubation and the washing were similar and non-significant to each other. It was interesting to observe that HaCaT keratinocytes did not display any signs of attachment, spreading, or aggregation when cultured on GelMA surfaces, unlike the DP cells, which exhibited signs of attachment and proliferation when cultured on GelMA surfaces. GFP-expressing DP and RFP-expressing HaCaT cells were used to visualize their orientation and compartmentalization within the microwells. It was observed that a core-shell configuration was achieved in the heterotypic spheroids seeded inside PEGDA microwells, with the DP aggregates forming the core and the HaCaT keratinocytes forming the shell on day 1. However, we also observed signs of compaction and migration of the DP cells towards the side of the microwells, accompanied by the exfoliation of the HaCaT keratinocytes on day 4 and day 7 of the co-culture. On the contrary, the sizes of the heterotypic spheroids cultured within GelMA microwells remained relatively constant throughout the seven days of incubation, with the DP cells mainly gathering at the base and the walls of the microwells and the HaCaT keratinocytes at the center, surrounded by the DP cells. Little to no signs of exfoliation of the HaCaT keratinocytes were observed in the heterotypic spheroids cultured within GelMA microwells.

Hair follicle development and cycling are controlled by EMI [23,24,25]. Hence, effective hair follicle models should permit interactions between epithelial and mesenchymal cells. Numerous molecules participate in the signaling exchanges during EMI. *Wnt10a* and *Wnt10b* are considered the ‘first epithelial signals’ at work during hair follicle morphogenesis [22]. *Wnt10a* has been reported to be expressed by the mesenchymal condensate at anagen onset, and *Wnt10b* is expressed in epithelial cells during the anagen phase [22]. Shh is expressed in the epithelial cells during morphogenesis, regulating proliferation and further downgrowth of the follicular epithelium and development of the DP [26]. From our gene expression analysis, the morphogens were clearly upregulated in the 3D co-cultures within PEGDA microwells as compared to GelMA on day 1 of incubation. We postulated that the aggregation of the DP may play a significant role in the upregulation of these morphogens responsible for EMI. PEGDA hydrogel provides a good low attachment substrate which encourages DP aggregation to partially restore DP inductive capacities through the upregulation of DP associated inductive genes and pathways, namely the *WNT*, *BMP*, and *FGF* signaling pathways [27]. However, the trend was reversed overtime (i.e., from day 4 to day 7), as the expression of these morphogens were similar or higher in co-cultures within GelMA microwells as compared to PEGDA. This is primarily due to the adherent nature of the DP cells which allowed the DP cells to remain attached to the base and walls of the GelMA microwells. Furthermore, little to no exfoliation was observed with the HaCaT keratinocytes within the GelMA microwells as compared to those cultured inside PEGDA microwells. The sustained presence of these cells within GelMA microwells, therefore, allowed for better EMI and, hence, the higher gene expression of the morphogens overtime.

The HDP and HaCaT cells used in both the PEGDA and GelMA systems are the same cells. By doing so, it removes any observed differences alluding to the cells and therefore, to the donor themselves. Any differences observed in the gene expression of the co-cultures (i.e., HDP and HaCaT) in both the PEGDA and GelMA systems can be ascribed to the differences in the intercellular interaction/aggregation, due to differences in cell-substrate and cell-cell interactions.

For future studies, immunofluorescence can be used to explore the influence of more morphogens, including *Wnt10A*, *Wnt10B*, and *Shh* on *in vitro* models for hair follicular engineering. In addition, the concentrations of PEGDA and GelMA in the hydrogels can be varied to study their potential effects on DP-HaCaT interactions.

## 4. Conclusion

We demonstrated that two different configurations of 3D heterotypic spheroids can be prepared using PEGDA and GelMA hydrogels by sequential seeding, due to differences in the inherent adhesiveness of DP and HaCaT keratinocytes towards these two different hydrogels. PEGDA microwells were able to produce a core-shell configuration with DP cells and HaCaT keratinocytes but this was not sustainable, due to poor intercellular adhesion between HaCaT keratinocytes. GelMA microwells were able to produce heterotypic aggregates over prolonged culture duration, which did not face the problem of exfoliating HaCaT keratinocytes. Future study would require greater in-depth study of how current GelMA hydrogel microwells could be improved to mimic hair follicle microenvironments for future in vitro hair follicle engineering pursuits.

## 5. Materials and Methods

### 5.1. Materials

Dulbecco’s modified Eagle’s medium (DMEM), fetal bovine serum (FBS), random primers, and SYBR safe DNA gel stain were supplied by Invitrogen, Life Technologies (USA). Trypsin and penicillin/streptomycin solution were obtained from PAN-Biotech GmbH (Germany). RNeasy Mini Kit and QuantiFast SYBR Green PCR kit were purchased from Qiagen (Germany). Random primers and avian myeloblastosis virus reverse transcriptase were purchased from Promega (Madison, WI, USA).

### 5.2. Master Fabrication

Photomasks were designed using AutoCAD 2010 and printed on chromium-coated soda lime glasses at Infinite Graphics PTE LTD (Singapore). Silicon wafers were spin-coated with negative photoresist SU-8 2075 (MicroChem Corp., Newton, MA, USA) at 1000 rpm, to give the desired film thickness of about 200 µm. Wafers were soft-baked at 65 °C for 7 min, followed by another soft-baking at 95 °C for 60 min. To crosslink the photoresist, coated wafers were exposed to UV light of 350–400 nm for 90 s through the photomask by using a single-side mask aligner (SVC, Model H94-25). Thereafter, the wafers were post-exposure baked at 65 °C for 6 min and then at 95 °C for 15 min. The photoresist-patterned silicon masters were developed using SU-8 developer, rinsed with isopropyl alcohol for 10 s, and air dried with pressurized nitrogen.

### 5.3. PDMS Stamp Fabrication

Prepolymer siloxane elastomer base solution was added to and mixed with curing agent Sylgard 184 (Dow Corning Corporation, Midland, MI, USA) at a 10:1 ratio by mass. The PDMS prepolymer mixture was poured onto a silicon master with a SU-8 photoresist coating patterned with an array of 200 µm microwells and degassed for 20–25 min in a vacuum chamber to eliminate any air bubbles before curing at 70 °C for 2 h. The PDMS layer was removed from the silicon master and cut to a suitable size. The resulting PDMS stamp had patterns corresponding to the silicon master in the form of micropillars with diameters of 200 µm each, and the micropillars on the PDMS stamp were imaged using a stereomicroscope (Nikon SMZ25, Tokyo, Japan).

### 5.4. GelMA synthesis

Gelatin (type A from porcine skin, bloom strength 300), Methacrylic Anhydride (MA) (contains 2000 ppm topanol A as inhibitor, 94%), and dialysis tubing (MWCO 12,400) were purchased from Sigma. All reactions were carried out at 50 °C, 10 grams of gelatin were dissolved in 80 mL deionized water. Sodium hydroxide 1.2 M was used to adjust the pH of clear gelatin solution to 9, then 6 mL of MA were added dropwise into the hot gelatin solution (at 50 °C) under vigorous stirring. After 3 hours of reaction, 200 mL of deionized water was added to stop the reaction, then all of the solution was put into dialysis tubing dipped in plenty of hot deionized water (~5 L) for 5 days. The water was changed every day. After dialysis, the gel solution was lyophilized for 5 days and stored in –80 °C.

### 5.5. PEGDA and GelMA Microwell Array Fabrication

UV-photocrosslinkable GelMA synthesized earlier or PEGDA (Jenkem Technology, USA) of molecular weight 3500 Da was mixed with photoinitiator Irgacure 2959, HHEMP (Sigma-Aldrich, USA) and diluted with 1× PBS to form a prepolymer solution containing the photoinitiator. The patterned PDMS stamp was placed on an evenly distributed film of prepolymer solution on a TMSPMA (Sigma-Aldrich, New York, NY, USA)-treated cover slip, with 2 coverslips set on both sides as spacers. Photopolymerization was attained by irradiating the set-up with UV light of 320–500 nm and at an intensity of 4.96 W/cm^2^ for 30 s using the OmniCure®Series 2000 curing station (Lumen Dynamics, Canada) as previously optimized. After photopolymerization, the PDMS stamp was removed from the fabricated hydrogel microwell arrays, which were submerged in 70% ethanol for 2 h to remove excess prepolymer solution. Hydrogel microwell arrays were then washed thrice with PBS and stored in sterile PBS under aseptic conditions prior to cell seeding.

### 5.6. Cell Culture

The immortalized DP cell line was donated by Professor Mike Philpott and Dr Adiam Bahta from Queen Mary University London for this work. The cell lines were previously extracted and immortalized from DP cells obtained from scalp biopsy [28]. DP, HaCaT keratinocyte cells, and HDF were manipulated under aseptic conditions and maintained in a humidified incubator at 37 °C with 5% CO_2_ atmosphere. Media components were filtered through 0.22 μm pore Corning filter units (Corning Incorporated, USA). Culture media consisted of DMEM (Invitrogen Corporation, USA) supplemented with 10% FBS (Invitrogen Corporation, USA), 1% 10,000 U/mL penicillin, and 10 mg/mL streptomycin (PAN-Biotech GmbH, Germany).

### 5.7. Preparation of 3D Cell Cultures

DP or HaCaT spheroids were prepared by seeding DP or HaCaT cell suspension at a concentration of 12 million cells per ml or 24 million cells per ml respectively into microwells by a previously reported wiping method [29]. Briefly, 15 µL of cell media (containing a certain concentration of cells) were pipetted along the edge of a microscopy glass coverslip which was then slowly wiped across a microwell array. The coverslip was wiped across the array at 1.0 mm/s and the array was placed in a humid enclosure to avoid evaporation of the isolated droplets in the microwells. HaCaT spheroids form 24 h after incubation at 37 °C and 5% CO_2_. To prepare the DP-HaCaT co-culture spheroids, DP cells were first seeded into microwells (concentration of cell suspension at 12 million cells per ml) by previously reported wiping method [29]. The DP cells were then incubated for 24 h for aggregate formation. HaCaT cells were subsequently seeded into the microwells on top of the DP aggregates using the same wiping method (concentration of cell suspension 24 million cells per mL) for co-culture. Co-cultured samples were then incubated in a humidified incubator at 37 °C and 5% CO_2_.

### 5.8. Cell Attachment Assay

Different wells from a 24 well plate (Greiner bio-one, Austria) were coated with 300 µL of 5% PEGDA or 5% GelMA and cured under UV light of 320–500 nm and at an intensity of 4.96 W/cm^2^ for 30 s using the OmniCure®Series 2000 curing station (Lumen Dynamics, Canada). Wells which were not coated with either PEGDA or GelMA were used as a control. These are wells from normal, non-treated wells in the 24 well plate. DP cells were seeded at 50,000 cells into the respective wells and incubated for 24 h. Non-adherent cells were washed away with PBS at 24 h after cell seeding. Attached cells were detached by trypsin/EDTA and counted by use of a hemocytometer in 20 µL per sample.

### 5.9. Cell Distributions in 3D Cultures

Lentiviral supernatant is obtained by co-transfecting 293T cells with pLenti-GFP [or pLenti-RFP] and ViraPower™ Lentiviral Expression System (Invitrogen, Singapore) according to the manufacturer’s instructions. DP and HaCaT cells were seeded at 50,000 cells/well in a 35 mm culture dish, respectively. Cells were cultivated in 37 °C, 5% CO_2_ humidified incubator overnight. DP cells were infected with GFP lentivirus for 48 h while HaCaT cells were infected with RFP lentivirus for 48 h. GFP-expressing DP cells and RFP-expressing HaCAT cells were sorted and isolated from non-transfected cells using flow cytometry with the instrument Beckman Coulter Moflo Astrios. Positively transfected cells were cultured and used in the preparation of DP-HaCAT co-cultures using the method as described above.

### 5.10. mRNA Extraction and Quantitative Real-Time PCR

mRNA was extracted from cells using the RNeasy Mini Kit (Qiagen, Germany) in accordance with the manufacturer’s instructions. Reverse transcription of total mRNA was performed using random primers and avian myeloblastosis virus reverse transcriptase. Quantitative Real-Time PCR reaction was performed using Rotor-Gene Q Real-Time PCR cycler (Qiagen, Hilden, Germany). Primers (Integrated DNA technologies, Singapore) used for PCR reactions were listed as follows in Table 1.

The primer sequences were designed using Primer3 (http://frodo.wi.mit.edu/) and Primer-BLAST (http://www.ncbi.nlm.nih.gov/tools/primer-blast/). GAPDH primer was included as an internal loading control. Each reaction mixture was prepared using 10 μL QuantiFast SYBR Green PCR master mix, 4 μL of cDNA template with 1 μM of each primer in a total reaction volume of 20 μL. The PCR was run for 40 cycles and the thermal cycling conditions were as follows: initial heat activation at 95 °C for 10 min; denaturation for 10 s at 95 °C; combined primer annealing and extension for 60 s at 60 °C. The fluorescence signal was measured at the end of each extension step. After the amplification, a melting peak analysis with a temperature gradient from 72 °C to 95 °C was performed. Fluorescence emission readings were analyzed using Rotor-Gene Q software (Qiagen, Germany). The data were presented as the fold increase of the target gene expression, normalized to the housekeeping gene GAPDH.

### 5.11. Statistical Analysis

Results were expressed as the mean ± standard deviation of at least three independent experiments. Statistical analysis was performed by one-way analysis of variance (ANOVA) and student’s *t*-test. The difference was statistically significant at *p*-value < 0.05.

## Figures and Tables

**Table 1:** DNA sequence of primer pairs used for quantitative real time PCR.

Gene	Forward Primer (5’- 3’)	Reverse Primer (5’- 3’)
GAPDH	TCGTGGAAGGACTCATGACC	ATGATGTTCTGGAGAGCCCC
*Shh*	GAGTCTCTGCACTACGAGGG	GCAGTGGATATGTGCCTTGG
*Wnt10A*	TCCATCCTTTCACCCCTTCC	GCTTCCATTCATTCCCCACC
*Wnt10B*	GTCACTCTTGGTCCCTGGAA	CCCCTCAAGACCCTCCAATT

## Data Availability

Not applicable.
